# Association of the *IL1RN* Gene VNTR Polymorphism with Human Male Infertility

**DOI:** 10.1371/journal.pone.0051899

**Published:** 2012-12-14

**Authors:** Deepika Jaiswal, Sameer Trivedi, Rajendra Singh, Rima Dada, Kiran Singh

**Affiliations:** 1 Department of Molecular and Human Genetics, Banaras Hindu University, Varanasi, India; 2 Department of Urology, Institute of Medical Sciences, Banaras Hindu University, Varanasi, India; 3 Division of Endocrinology, Central Drug Research Institute, Lucknow, India; 4 Department of Anatomy, All India Institute of Medical Sciences, New Delhi, India; Sudbury Regional Hospital, Canada

## Abstract

Interleukin-1 (IL-1) is a regulatory cytokine that plays an important role in the maintenance of the immune environment of the testis, regulation of junction dynamics and cell differentiation during spermatogenesis. Members of the IL-1 family are pleiotropic cytokines that are involved in inflammation, immunoregulation and other homeostatic functions in the body. IL-1α, IL-1β, and the IL-1 receptor antagonistic molecule (IL-1 Ra) are expressed in the testis under normal homeostasis and they further increase upon infection/inflammation. In the present study we have examined the association of Variable Number Tandem Repeats (VNTR) polymorphism of the Interleukin-1 receptor antagonist gene (*IL1RN*) with human male infertility. The case-control study comprised of two groups: 331 idiopathic infertile patients and 358 fertile healthy men. The study indicates risk of *IL1RN2* variant with male infertility (OR: 1.43, CI: 1.1546 to 1.7804, P = 0.001). To our best knowledge, this is the first report that links *IL1RN* VNTR polymorphism with human male infertility.

## Introduction

Testicular homeostasis requires a unique immune status within the male gonad. The immune system acts as a safeguard for immunogenic male germ cells and at the same time permits normal inflammatory response against invading pathogens [1]. Most events of spermatogenesis take place in a unique microenvironment behind the blood-testis barrier (BTB), which is created between adjacent Sertoli cells near the basement membrane of the seminiferous tubule. Cytokines mediate the crosstalk between Sertoli and germ cells to facilitate germ cell movement across the seminiferous epithelium during cellular events such as germ cell differentiation. IL-1α, IL-1β, and the IL-1 antagonistic molecule (IL-1 Ra) are present in the testis under normal homeostasis and they further increase upon infection/inflammation [2]. Interleukin-1 was also shown to be involved in the regulation of junction dynamics during spermatogenesis [3]. Recently, it was demonstrated that knockout of IL-1 receptor antagonist gene (*IL1RN*) in mouse affects the normal fertility of males [4]. The variable number tandem repeats (VNTR) polymorphism has been reported within intron 2 of the human *IL1RN*, consisting of perfect repeats of 86- bp sequence [5]. The number of repeats is of functional significance as these repeats contain binding sites for transcription factors [6]. Therefore, we hypothesized that VNTR polymorphism of *IL1RN* gene may influence spermatogenesis and thereby fertility. Based on the biological and pathologic importance of (VNTR) polymorphism of the human *IL1RN*, it is possible that variations in the *IL1RN* gene may contribute to the clinical outcomes of male infertility.

## Materials and Methods

In the present case-control study subjects were recruited from the same geographical region i.e. Northern part of India (Delhi, Lucknow and Varanasi) and they all belong to the same ethnic group. The study was approved by the Institutional Human Ethics Committee of Institute of Medical Sciences, Banaras Hindu University, Varanasi, India. Informed written consent was obtained from every participant of each group. To examine this hypothesis, we analyzed the VNTR of the *IL1RN* gene in 331 male infertility cases and in 358 healthy fertile controls as previously described [5]. Patients married for a minimum of two years, having unprotected intercourse were considered for the present study. Three semen analyses were carried out after three/four days of sexual abstinence to ascertain their infertility status. The patients were categorized in sub-groups as per WHO 1999 criteria. Asthenozoospermic infertile men (N = 69) had a sperm count >20x10^6^/mL, motility< = 50%, and> = 30% normal morphology, Severe Oligozoospermia infertile men had sperm count <2x10^6^/mL and Oligozoospermia infertile men had sperm count <10x10^6^/mL (normal motility and morphology) and non-obstructive azoospermic infertile men (N = 141) with no sperm in the ejaculate. Patients with obstructive azoospermia, hypogonadism, hypoandrogenism, chronic diseases, history of pelvic/spinal injuries, karyotype abnormalities and AZF microdeletions were excluded. The control group consists of healthy fertile males who have at least one child and no history of chronic illness.

### *IL-1RN VNTR* (86-bp Repeat in Intron 2)

*IL-1RN* gene polymorphism was analyzed as previously described [7]. The polymorphic region was amplified by PCR: 30 cycles (40 s at 94°C, 40 s at 57°C, 40 s at 72°C) with primers 5′-CTCAGCAACACTCCTAT-3′ and 5′-TCCTGGTCTGCAGGTAA-3′. The PCR products were analyzed by 2.5% agarose gel electrophoresis. *IL-1RN1* allele corresponded to a 410-bp fragment (four copies of the 86-bp repeat), *IL-1RN2* to a 240-bp fragment (two copies), *IL-1RN3* to a 325-bp fragment (three copies), *IL-1R4* to a 500-bp fragment (five copies), *IL-1RN5* to a 585-bp fragment (six copies) and *IL-1RN6* to a 155-bp fragment (one copy).

### Statistical Analysis

Allele and genotype distribution between groups were evaluated using Chi-square test or Fisher Exact test. The difference in frequencies between the case and control groups was analyzed for statistical significance at the 95% confidence interval using χ^2^ test and Yates’s correction. The allele frequency of IL-Ra was in Hardy-Weinberg equilibrium. Odds ratios (ORs) were calculated and reported within the 95% confidence limits. A p-value of <0.05 was considered as significant in all the analyses.

## Results

**Figure 1:**
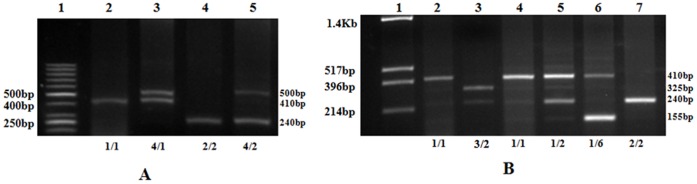
PCR amplified products of IL1RN genes VNTR polymorphism. A. Lane 1 ∶50 bp DNA ladder; **Lane 2**: *IL1RN1*/*IL1RN1*; **Lane 3**: *IL1RN1/IL1RN4*, **Lane 4:** *IL1RN2/IL1RN2* **, Lane5:** *IL1RN4/IL1RN2*. **B. Lane 1**: puc/hinf1 DNA ladder; **Lane 2:** *IL1RN1/IL1RN1* **, Lane 3:** *IL1RN2/IL1RN3* **, Lane 4:** *IL1RN1/IL1RN1* **, Lane5:** *IL1RN1/IL1RN2*, **Lane 6:** *IL1RN1/IL1RN6* **, Lane 7:** *IL1RN2/IL1RN2*.

**Figure 2:**
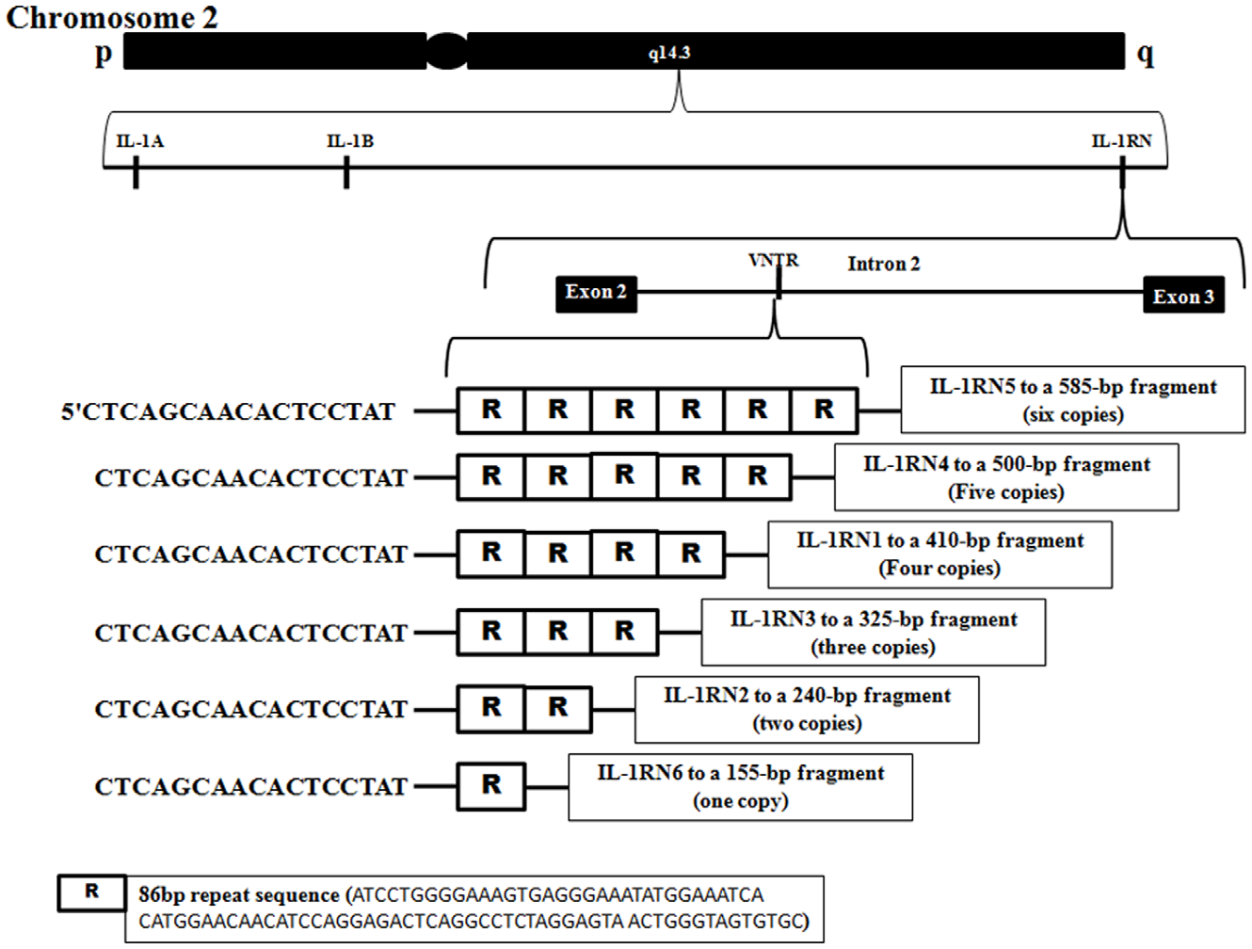
Schematic diagram of the IL1RN gene showing the position of the VNTR polymorphism along with various alleles.

Five allelic variants of the *IL1RN* gene with variable number of repeats from one to six have been identified (Fig 1 and Fig 2). DNA sequencing was used to establish VNTR copy number. The allele frequency of *IL1RN* was in Hardy-Weinberg equilibrium. The genotype and allele frequencies for VNTR in the *IL1RN* gene were compared between controls and different groups of patients (Table 1). The statistically significant allelic association was detected only between *IL1RN2* allele and male infertility (P = 0.001). Thus, *IL-1RN2* VNTR polymorphism confers genetic susceptibility to human male infertility. This is the first report which observed sixth allele in the Asian population.

**Table 1:** Distribution of VNTR of *IL1RN* genotypes and alleles among infertile patients and fertile controls.

Genotypes	Controls	Azoospermia(1)	Severe Oligozoospermia/ oligozoospermia	Asthenozoospermia	Total	Odds Ratio (95% CI^)#^				p-Value#				Yates’ Corrected p-Value#			
	(n = 358)	(n = 168 )	(2) (n = 42 )	(3) (n = 121)	(1+2+3) (n = 331)	(1)	(2)	(3)	Total (1+2+3)	(1)	(2)	(3)	Total (1+2+3)	(1)	(2)	(3)	Total (1+2+3)
IL1RN1/1	155 (43.29%)	55 (32.73%)	16 (38.09%)	37 (30.58%)	108 (32.63%)	Reference				–							
IL1RN2/2	60 (16.76%)	38 (22.61%)	9 (21.43%)	33 (27.27%)	80 (24.17%)	1.8 (1.0765 to 3.0544)	1.5 (0.5947 to 3.6936)	2.4 (1.3468 to 4.2469)	1.9 (1.2635 to 2.8718)	0.02*	0.39	0.002*	0.002*	0.03*	0.42	0.004*	0.003*
IL1RN1/2	139 (38.83%)	69 (41.07%)	13 (30.95%)	49 (40.49%)	131 (39.58%)	1.4 (0.9182 to 2.1236)	0.9 (0.4226 to 1.9446)	1.5 (0.9112 to 2.3795)	1.4 (0.9605 to 1.9001)	0.11	0.80	0.11	0.08	0.14	0.95	0.14	0.1
Rare##	4 (1.12%)	6 (3.57%)	4 (9.52%)	2 (1.65%)	12 (3.62%)	5.4 (1.3042 to 22.0525)		2.4 (0.3156 to 18.5884)	3.9 (1.4361 to 10.9908)	0.01*	0.003*	0.39	0.007*	0.04*	0.003*	0.74	0.01*
IL1RN alleles																	
IL1RN1	452 (63.13%)	183 (54.46%)	48 (57.14%)	125 (51.65%)	356 (53.77%)	Reference				–							
IL1RN2	260 (36.31%)	147 (43.75%)	32 (38.09%)	115 (47.52%)	294 (44.41%)	1.4 (1.072 to 1.8301)	1.2 (0.7194 to 1.8742)	1.6 (1.1945 to 2.1733)	1.4 (1.1546 to 1.7804)	0.01*	0.54	0.001*	0.001*	0.02*	0.62	0.002*	0.001*
Rare##	4 (0.56%)	6 (1.78%)	4 (4.76%)	2 (0.83%)	12 (1.81%)	4.5 (1.1405 to 17.7115)	-	1.9 (0.2828 to 13.882)	3.5 (1.2952 to 9.473)	0.03*	0.0001*	0.49	0.01*	0.07	0.001*	0.84	0.03*

Note: OR =  odds Ratio; CI = 95% confidence interval.

* Significant p-value;

# Controls *vs.* (1), (2), (3), (1+2+3).

## Rare genotypes contain alleles 3, 4, or 6.

There is no simple link between the number of repeats and the number of the allele.

## Discussion

The cytokine network helps in synchronizing multifaceted communication during spermatogenesis [3]. Cytokines are secreted in the semen plasma which indicates their role in fertilization and pregnancy [5]. Elevated levels of cytokines in the seminal plasma of infertile men have been well documented [8]. The cytokine, Interleukin 1 (IL-1) includes family of three proteins: IL-1 alpha, beta and IL-1Ra [9]. IL-1 modulates the expression of genes involved in cell-survival, proliferation, and angiogenesis processes important for spermatogenesis. IL-1 Ra is a natural receptor antagonist for IL-1gene and functions as a negative regulator of IL-1 by inhibiting a positive feedback loop in which IL-1 induces its own production thus affect its function [10], [11]. *IL1RN* VNTR polymorphism alters IL-1 levels involved in regulation of spermatogenesis, fertilization and pregnancy outcomes. Rozwadowska et al, have reported that the ratio of IL-1α to IL-1 Ra is altered in cases of azoospermia with impaired spermatogenesis and hence affect male fertility [12]. Both alpha and beta interleukin 1 is produced by testicular Sertoli cells [13] and through their activation they inhibit the production of testosterone that is stimulated by luteinizing hormone, human chorionic gonadotrophin and cAMP [14], [15]. In absence of testosterone cell apoptosis is greatly increased that in turn have negative impact on spermatogenesis [16]. In another report Gopichandran et al, have examined the role of numerous cytokines present within the seminal plasma in immunomodulation, uterine leukocyte recruitment and trafficking [17]. The VNTR polymorphism in *IL1RN* affects the expression of IL-1Ra [18] suggesting that the levels of IL-1 could be affected that in turn could disturb the normal physiology in the testis. Another evidence from *IL1RN* knockout mouse model shows that the knockout mice have higher levels of IL-1 in the testis and the IL1RN-deficiency leads to male infertility in these mice due to impaired ability of sperm to fertilize oocyte [4]. With above discussion the association of VNTR polymorphism can be explained to be associated with both azoospermia and normoasthenozoospermia. Because of altered IL-1 levels if spermatogenesis is affected it may give rise to azoospermic phenotype whereas if sperm function is affected as in case of IL-1Ra knockout, it may give rise to asthenozoospermia phenotype which have an impact on fertilization and further pregnancy outcomes. This is first report on the presence of rare single copy alleles (*IL1RN6)* in Asian populations and association of *IL1RN2* allele with human male infertility. The IL-1 gene family is expressed in the male gonad and the profile of its expression can be a promising predictive marker of spermatogenetic impairment.

## Supporting Information

Appendix S1 Sample of a patient consent form.
(DOC)
