# Supplementary material for: Association of the IL1RN Gene VNTR Polymorphism with Human Male Infertility
Source: PLoS One. 2012 Dec 14;7(12):e51899. doi: 10.1371/journal.pone.0051899 (PMC3522614; doi:10.1371/journal.pone.0051899)
Supplement: Appendix S1 — Sample of a patient consent form. (DOC) [file pone.0051899.s001.doc]

**Sample of a patient consent form**

I have been advised to undergo molecular genetics/chromosomal test for myself by my consultant doctor for the analysis of the suspected genetic defects.

I hereby give consent to provide the sample of blood for the above said diagnosis. I understand that the result obtained will be disclosed only to me and my consultant. I also agree that obtained results will be obtained from these can be used for scientific publication under the confidentiality of my name.

Signature:

Name and Address of the signatory:

Age:

Place and Date:

Signature of the two witnesses:
